# Perinatal Management Pathways for Transposition of the Great Arteries With Intact Ventricular Septum: A National Survey

**DOI:** 10.1111/1471-0528.18281

**Published:** 2025-07-07

**Authors:** Victoria Jowett, Anna N. Seale, Julene S. Carvalho, Lindsey Hunter, Trisha Vigneswaran, Shuba Barwick, David Black, Patricia Caldas, Orla Franklin, Caroline Jones, Sophia Khan, Katie Linter, Alexandra Matthews, Magdalena Sajnach‐Menke

**Affiliations:** ^1^ Cardiology Department Great Ormond Street Hospital London UK; ^2^ Institute of Cardiovascular Science University College London London UK; ^3^ Paediatric Cardiology Department Birmingham Women's and Children's Hospital Birmingham UK; ^4^ Paediatric Cardiology Royal Brompton Hospital London UK; ^5^ Fetal Medicine St Georges University Hospital London UK; ^6^ Paediatric Cardiology Royal Hospital for Children Glasgow UK; ^7^ Paediatric Cardiology Evelina Childrens Hospital, Guys and St Thomas' Hospital London UK; ^8^ Biomedical Engineering and Imaging Sciences Kings College London London UK

1

Transposition of the great arteries with intact ventricular septum (TGA‐IVS) is increasingly diagnosed antenatally [1] which allows for perinatal planning [2]. Outcomes for definitive surgery are excellent, but there is pre‐surgical morbidity and mortality resulting from neonatal hypoxia due to poor mixing through the atrial communication, restrictive or closed arterial duct, or a severe form of newborn persistent pulmonary hypertension (PPHN) [3]. Newborns with inadequate atrial mixing require urgent balloon atrial septostomy (BAS), in some cases as a lifesaving procedure, in the first hours of life. Risk can be stratified antenatally but is imperfect [2, 4].

We designed a questionnaire that was sent to all cardiac surgical units in the United Kingdom to obtain details of the perinatal pathway for patients with an antenatal diagnosis of TGA‐IVS (Appendix S1) The aim was to examine how individual units in the United Kingdom have sought to minimise the pre‐surgical risks in patients prenatally diagnosed with TGA‐IVS and to understand the constraints of geography and staffing, which could identify areas for future study to improve outcomes in this high‐risk group.

All 11 (100%) cardiac units in the United Kingdom and Ireland undertaking surgery for TGA‐IVS responded. Almost half of the units 5/11 (45%) have maternity and cardiac services co‐located (Figure 1). The distance from the maternity unit to the cardiac centre for non‐co‐located units ranges from 0.7 to 3.6 miles. Final fetal cardiology assessment was at 32–34 weeks in 2/11 (18%), 34–36 weeks in 7/11 (63%) and > 37 weeks in 2/11 (18%).

**FIGURE 1:**
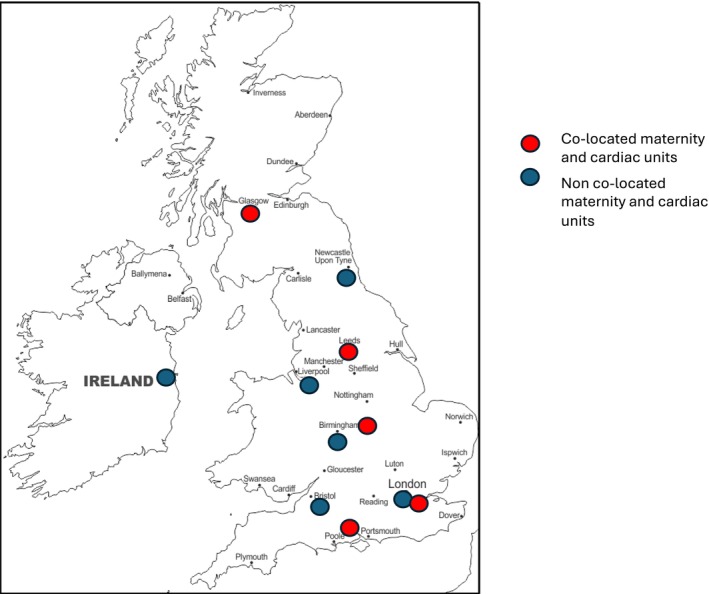
UK map showing cardiac units and co‐location with maternity units.

The planned mode of delivery, when there were no additional maternal obstetric concerns, was spontaneous labour in 3/11 (27%), induction of labour in 6/11 (55%) and elective caesarean section (ELCS) in two units (18%) both of which were non‐co‐located units. One additional co‐located unit and two additional non‐co‐located units delivered by elective C section if there was concern that the foramen ovale (FO) was small prenatally. The planned week of delivery was 38 weeks for all non‐co‐located units. For co‐located units, 2/5 planned delivery at 38 weeks and 3/5 at 39 weeks. Units had two to four interventional cardiologists. It was not standard practice at any unit to have an interventional cardiologist present for every delivery of a baby with TGA‐IVS. Two units stated that an interventional cardiologist is present at delivery when there was concern of a small FO. Emergency septostomy was performed in the catheter lab in 3/5 co‐located units and in 3/6 non‐co‐located units, whereas 5/11 (45%) would perform this at the bedside or in obstetric theatre. Seven units reported a change in their practice in 2019 following the manufacturers recall of the Millar Edwards septostomy catheter, which resulted in the need to use an over a guidewire septostomy balloon, licensed for catheter lab use. Three units initially changed to performing in the catheter lab but subsequently reverted to bedside septostomy.

Two units are able to accommodate patients before delivery when their home is geographically distant from the maternity unit.

Whilst co‐location of maternity and cardiac services provides an advantage in improving access to emergency septostomy and reducing transport risks, logistical constraints such as funding and hospital infrastructure prevent co‐location in all units. This survey highlights the variation in practice around the United Kingdom of perinatal pathways developed by units to mitigate risk according to their local infrastructure and resources. Notably, some non‐co‐located units would transfer newborns from the maternity unit to the cardiac centre in order to perform an emergency septostomy, which may increase time to septostomy. Fetal echocardiography can identify some, but not all patients who will require an emergency septostomy; this risk needs to be managed. The increased rate of delivery by ELCS in non‐co‐located units suggests that these units have managed the risk by more precise control of delivery time offered by ELCS, but this adaptation is not free from complications. A greater proportion of non‐co‐located units planned delivery at 38 rather than 39 weeks' gestation. Although earlier delivery may reduce the risk of delivery outside of a controlled setting, this needs to be balanced against neonatal risks such as increased need for respiratory support in early term neonates, impact of reduced birth weight on surgical outcomes, longer length of stay for infants born at earlier gestation [5] and adverse impact on neurological outcome [6]. There were no units which routinely allocated an interventional cardiologist to be present at the time of delivery, and this may reflect staffing resources.

The increase in antenatal diagnosis of TGA‐IVS provides an opportunity to improve the perinatal management. All units delivering the high‐risk neonate with TGA‐IVS require a pathway that can facilitate emergency balloon atrial septostomy when needed. Without further studies, at the current time, it is not possible to be prescriptive about what constitutes safe practice.

Further research is needed to compare different models of care to identify best practice and highlight variability for future standardisation. This would include establishing the impact of co‐location, the optimal mode and timing of delivery, and prenatal predictors for hypoxia in this high‐risk group.

## Author Contributions

The study was conceived, planned and carried out by V.J.; all authors and those acknowledged from the national fetal cardiology working group and BCCA contributed to the completion of the survey. A.N.S., J.S.C., L.H. and T.V. contributed to the writing of the research letter.

## Conflicts of Interest

The authors declare no conflicts of interest.

## Supporting information


Appendix S1.

## Data Availability

The data that support the findings of this study are available from the corresponding author upon reasonable request.
