# Supplementary material for: Perinatal Management Pathways for Transposition of the Great Arteries With Intact Ventricular Septum: A National Survey
Source: BJOG. 2025 Jul 7;133(1):178–80. doi: 10.1111/1471-0528.18281 (PMC12676189; doi:10.1111/1471-0528.18281)
Supplement: Supplementary file 1 — Appendix S1. [file BJO-133-178-s001.docx]

**Supplementary material - TGA-IVS National Survey - questions**

1. Are TGA IVS patients delivered on the same site as the cardiac unit?

•         If no, where are they delivered and how far (distance and approximate minutes travel) is it between the cardiac unit and delivery unit?

1. Are TGA IVS delivered by:

(a) Awaiting spontaneous delivery

(b) Induction of labour

(c) elective C section

Please include if differences for different groups eg primips/multips/fetal impression of restrictive septum

At what gestation for IOL and C section be planned (in the absence of maternal obstetric concerns)

1. Are your more geographically distant patients accommodated locally in the run up to delivery and if so from what gestation.
2. How many interventional cardiologist do you have

1. Is the interventional cardiologist:
   1. present at the time of delivery or
   2. called if needed.
2. Have you made any changes to the perinatal management of TGA following the withdrawl of the(Miller-Edwards) septostomy balloons?
3. Is balloon atrial septostomy performed
   1. at the bedside or
   2. in the cath lab

1. How many weeks before delivery would you perform your final fetal assessment of the atrial septum?
2. What percentage of patients have BAS pre arterial switch procedure (if data available)
